# Gene expression changes in the cerebellum are associated with persistent post-injury pain in adolescent rats exposed to early life stress

**DOI:** 10.1016/j.ynpai.2023.100145

**Published:** 2023-11-08

**Authors:** Sabrina Salberg, Crystal N. Li, Jaimie K. Beveridge, Melanie Noel, Glenn R. Yamakawa, Richelle Mychasiuk

**Affiliations:** a Department of Neuroscience, Monash University, Melbourne, VIC, Australia; b Department of Psychology, Alberta Children’s Hospital Research Institute, Hotchkiss Brain Institute, The University of Calgary, Calgary, AB, Canada

**Keywords:** Adverse Childhood Experiences, Nociception, RT-qPCR, CGRP, Substance P

## Abstract

Chronic pain develops following injury in approximately 20% of adolescents, at twice the rate in females than males. Adverse childhood experiences also increase the risk for poor health outcomes, such as chronic pain. Emerging literature suggests the cerebellum to be involved in pain processing, however detailed explorations into how the cerebellum contributes to pain are lacking. Therefore, this study aimed to characterise chronic pain outcomes and cerebellar gene expression changes following early life stress and injury in both sexes. The adverse childhood experience of neglect was modelled using a maternal separation (MS) paradigm, which was combined with a subsequent injury (mild traumatic brain injury (mTBI) or plantar incision surgery) in adolescent male and female Sprague-Dawley rats. We measured behavioural nociceptive sensitivity, systemic modulators of pain such as calcitonin gene-related protein (CGRP) and Substance P, as well as gene expression of *IL1β*, *GFAP, GR, MR, GABRA1, CNR1, MAOA,* and *DAT1* in the cerebellum to examine associations between pain and neuroinflammation, the stress response, inhibitory neurotransmission, and monoaminergic function. We found increases in mechanical nociceptive sensitivity following plantar incision surgery. Sex differences were observed in anxiety-like behaviour and neuroinflammation, whereas systemic pain modulators showed cumulative effects with the addition of stressors. Most interestingly however, the increases in nociceptive sensitivity were associated with the suppressed expression of cerebellar genes that regulate stress, inhibition, cannabinoid function, and dopaminergic function, alongside sex-dependent distinctions for genes involved in inflammation and injury. This study highlights a novel link between nociception and molecular function in the cerebellum. Further investigation into how the cerebellum contributes to pain in males and females will facilitate novel therapeutic insights and opportunities.

## Highlights

• Pain outcomes and cerebellar gene expression were examined in adolescent rats.
• Similar behavioural pain outcomes were found in male and female rats following injury.
• Injury and stress manipulations reduced cerebellar expression of genes relevant to stress and neurotransmission.
• Injury and stress-dependent changes in gene expression were often sexually dimorphic.
• There is a link between injury-induced changes in pain and gene expression in the cerebellum.

## 1 Introduction

Chronic pain often arises in adolescence, at twice the rate in females than males (King et al., 2011). Although the aetiology of chronic pain is not well understood, numerous variables have been proposed to underlie its manifestation. Chronic pain can develop following an acutely painful stimulus or injury, with post-traumatic headache and post-surgical pain persisting in 20% of youth that sustain a mild traumatic brain injury (mTBI) (Eisenberg et al., 2014) or undergo a surgical procedure (Rabbitts et al., 2017), respectively. Adverse childhood experiences have also been shown to increase risk for poor mental and physical health outcomes, including chronic pain (Green et al., 2011, Beal et al., 2020, Noel et al., 2016). In line with these clinical findings, we have previously demonstrated that rodents exhibit increases in mechanical nociceptive sensitivity following a plantar incision surgery or a mTBI (Salberg et al., 2023). Furthermore, heightened mechanical sensitivity is also observed when rodents are exposed to a maternal separation (MS) paradigm for modelling the adverse childhood experience of neglect (Salberg et al., 2023). In examining the mechanisms associated with these nociceptive changes, our laboratory found that males with nociceptive hypersensitivity had increased inflammatory and macrophage activity, whereas females only demonstrated behavioural sensitivity, without exhibiting changes in those pathophysiological mechanisms (Salberg et al., 2023). Inflammation was observed systemically, as well as in the sensory, limbic, and prefrontal regions of the brain. While there is a broad consensus for the role of these regions in the acute sensory features, and chronic affective and cognitive aspects of pain (Brooks and Tracey, 2005, Apkarian et al., 2005, Hashmi et al., 2013), recent insights into additional brain regions involved in pain processing have emphasized the cerebellum (Moulton et al., 2010), a region that was not considered in the aforementioned study.

Early evidence documenting a role for the cerebellum in pain evolved from electrophysiological studies in animals demonstrating that direct afferents into the cerebellum were activated by stimulation of peripheral nociceptive fibres (Ekerot et al., 1987, Wu and Chen, 1992). Emerging literature from human neuroimaging studies consistently demonstrates activity changes within the cerebellum in experimental pain (Henderson et al., 2008, Svensson et al., 1997) and chronic pain (Moulton et al., 2010, Borsook et al., 2008, Youssef et al., 2014). Despite this, detailed explorations regarding how the cerebellum contributes to pain are lacking. While the cerebellum is well known for its role in sensorimotor processing, there have been additional considerations for its participation in the affective and cognitive facets of pain beyond mere sensorimotor aspects, however these remain largely speculative (Moulton et al., 2010, Grodd et al., 2001).

Moreover, although prior neuroimaging investigations have demonstrated pain-related changes to cerebellar activity at a regional level, very few have examined changes from a cellular or molecular basis. Here, animal models of pain provide valuable opportunities for analysis of tissue in ways that are not achievable in human studies. Therefore, this study aimed to characterise chronic pain outcomes and gene expression changes in the cerebellum following early life stress and injury in male and female adolescent rats. We combined a MS paradigm with a subsequent mTBI or plantar incision surgery and measured behavioural nociception and comorbid anxiety, as well as systemic modulators of pain such as calcitonin gene-related peptide (CGRP) and Substance P. Additionally, we examined gene expression changes in interleukin 1 beta (*IL1β*) and glial fibrillary acidic protein (*GFAP*) within the cerebellum for associations between neuroinflammation and pain. Glucocorticoid receptor (*GR*) and mineralocorticoid receptor (*MR*) expression were assessed for insights into the stress response. Moreover, considering the high density of GABAergic neurons and cannabinoid receptors in the cerebellum (Herkenham et al., 1990), expression of gamma-aminobutyric acid type A receptor subunit alpha1 (*GABRA1*) and cannabinoid receptor 1 (*CNR1*) were assessed, and gene expression of markers for monoaminergic function were also quantified due to their relevance in cerebellar modulation (Ossipov et al., 2014). We hypothesized that animals exposed to early life stress and injury paradigms would display increased nociceptive sensitivity and levels of circulating systemic pain modulators, as well as reduced cerebellar expression of genes involved in stress, inhibitory neurotransmission, and monoaminergic function; and that these changes would manifest in a sex-dependent manner.

## 2 Methods

### 2.1 Animals

**Fig. 1:**
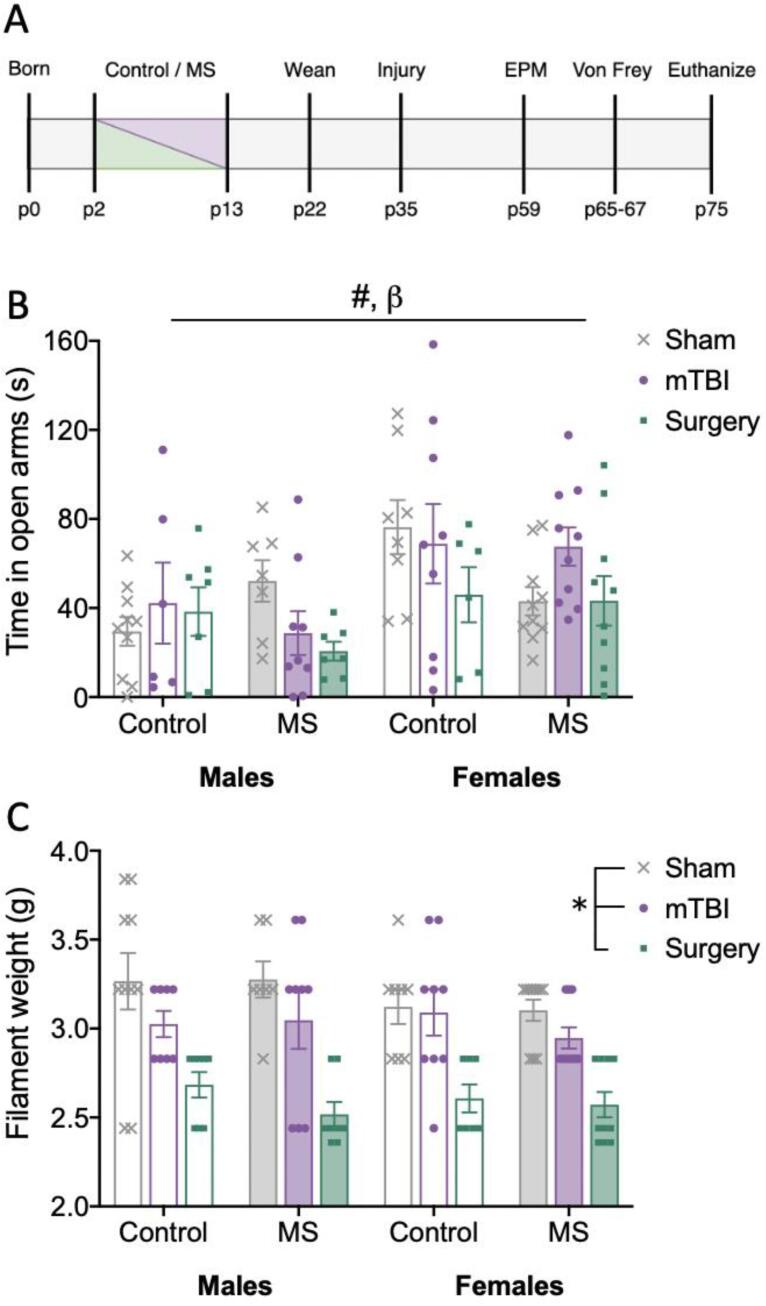
Experimental timeline and bar graphs displaying results for behaviour tasks. Means and individual data points ± standard error are shown. (*) indicates a main effect of injury, (#) indicates a main effect of sex, and (β) indicates a significant interaction; *p’s* < 0.05. A) Experimental timeline, MS – maternal stress, EPM – elevated plus maze; B) Time spent in the open arms of the elevated pus maze task, whereby the interaction observed was sex*stress*injury; and C) Filament weight recorded on the von Frey task for the right hind-paw.

All experiments were carried out in accordance with the Precinct Animal Centre (E/1928/2019/M) and ARRIVE guidelines and approved by the Alfred Medical Research and Education Precinct Animal Ethics Committee. Adult Sprague Dawley rats were acquired from the Monash Animal Research Platform and bred in-house, with male and female offspring being used for this study. All animals were maintained on a 12:12 hr light:dark cycle (lights on 0700) with *ad libitum* access to standard food and water in a temperature controlled (21°C) environment. Pups were randomly assigned to a control or MS group, then further into a sham, mTBI, or surgery condition (n = 7–10/group/sex). No more than two pups from any litter were assigned to a specific condition. The MS paradigm consisted of removing the pups from their mother for four hrs/day (1000–1400) for 12 consecutive days (p2-p13) as validated previously (Salberg et al., 2023, Salberg et al., 2021, Salberg et al., 2020). Pups were placed together as a litter in a separate, half-heated cage for the duration of the four hours, then returned to their mothers. The control group remained undisturbed with their mothers until weaning (p22). Following weaning, rats were group-housed (n = 3–4/cage) with cage mates from the same injury condition. See Fig. 1A for experimental timeline.

### 2.2 Injury

The mTBI procedure was induced using the lateral impact model, as previously described (Salberg et al., 2023, Mychasiuk et al., 2016). Briefly, rats were anesthetized with 5% isoflurane at 1 L/min O_2_ until unresponsive to a toe-pinch. A 50 g weight was then propelled towards the left temporal lobe, which first impacted an aluminum plate to prevent structural damage to the skull. The force caused the rat to rotate 180° at which point it was placed in a recovery cage and time-to-right was recorded. The surgery paradigm utilized the Brennan model, as previously described (Salberg et al., 2023, Brennan et al., 1996). Rats were anesthetized with 5% isoflurane at 1 L/min O_2_ until unresponsive, then transferred to a nosecone with 2–3% isoflurane at 1 L/min O_2_ for the duration of the procedure. The left hind-paw was sterilized with chlorhexidine and 70% ethanol and a ∼1 cm incision was made in the skin from the heel to the first footpad. The underlying plantaris muscle was then located and incised three times before the wound was closed with two simple interrupted sutures and cleaned. To encompass a sham procedure for both injury models, the sham procedure consisted of anesthetizing the rats with 5% isoflurane at 1 L/min O_2_ until unresponsive, followed by time-to-right observation in a recovery cage.

### 2.3 Behaviour

The current study sample includes a subset of animals from a distinct comprehensive study, with behaviour data including the additional groups published elsewhere (Salberg et al., 2023). All behaviours were carried out by researchers blinded to experimental conditions. The elevated plus maze (EPM) was conducted as a measure of anxiety-like behaviour (Walf and Frye, 2007), whereby the rat was placed in the centre of a plus shaped maze raised 51 cm from the ground. It consisted of two closed (enclosed with vertical walls) and two open arms (51 cm x 11 cm), intersecting in a centre area (11 cm x 11 cm). A single 5 min trial was run, with overhead TopScan software tracking the free movement of the rat. Increased time spent in the open arms was indicative of reduced anxiety-like behaviour. The von Frey test was used as a measure of mechanical sensitivity (Chaplan et al., 1994, Le Bars et al., 2001), whereby increasing size filaments were applied to the rats' hind-paws. The rats were placed inside boxes on top of a small wire grid (0.6 cm squares). Animals were habituated to the apparatus by being placed inside the boxes undisturbed for 20 min for two consecutive days. On the third day, animals were again placed inside the box for 20 min to habituate and then tested, at which point the increasing size filaments were applied to the right (uninjured) hind-paw of the rat 5 times. The number of responses (withdrawal of hind-paw) was recorded, and the test was stopped once a 5/5 reaction was observed, with the corresponding filament weight being recorded. The larger the filament weight, the higher the mechanical nociceptive threshold.

### 2.4 RT-qPCR and ELISAs

At euthanasia (p75), rats were anesthetized with isoflurane until unresponsive to a toe-pinch, which was followed by rapid guillotine decapitation, removal of the brain, and collection of trunk blood. The posterior cerebellum was collected, flash frozen, and stored at –80°C. RNA was extracted from 20 mg of cerebellum tissue using the RNeasy Mini Kit in conjunction with the QIAcube (Qiagen, Germany), according to manufacturer’s protocols. 2 µg of RNA were reverse transcribed to cDNA with qScript™ XLT cDNA SuperMix (Quantabio, USA) following manufacturer’s instructions. Each sample was run in duplicate on the QuantStudio (Thermo Fisher Scientific, USA) for RT-qPCR with 20 ng cDNA, 1 X SYBR Green FastMix ROX, and 0.5 uM of forward and reverse primers in each well. The 2^-ΔΔCt^ method was used for gene expression analysis, with the housekeeping genes *Ywhaz* and *Cyca* used for normalization (Pfaffl, 2001, Bonefeld et al., 2008). Analysis was performed by an investigator blinded to the experimental conditions of the samples. Two samples were excluded from analysis of *DAT1* as they failed to meet quality control requirements. Primers were obtained from Integrated DNA Technologies (USA), and primer sequences and cycling parameters can be found in Table 1. Blood was collected in serum separator tubes, allowed to clot at room temperature for 30 min, centrifuged, and the serum was aliquoted and stored at –80°C. Serum was used to run enzyme-linked immunosorbent assays (ELISAs) for CGRP and Substance P, according to the manufacturer’s protocols (Resolving Images, Australia).

**Table 1:** Primer sequences and cycling parameters for RT-qPCR.

**Gene symbol**	**Gene name**	**Primer sequence**	**Tm (°C)**	**Cycling parameters**
*CYCA*	Cyclophilin A	(+) agcactggggagaaaggatt (−) agccactcagtcttggcagt	60	1 cycle 95°C 20sec 40 cycles 95°C 1sec 40 cycles Tm°C 20sec + Melt Curve
*YWHAZ*	Tyrosine 3-monooxygenase	(+) ttgagcagaagacggaaggt (−) gaagcattggggatcaagaa		
*IL1β*	Interleukin 1 beta	(+) gggcctcaaggggaagaatc (−) atgtcccgaccattgctgtt		
*GFAP*	Glial fibrillary acidic protein	(+) cttgtttgctaggcccaattcc (−) atttgttaagtctccctgcccc		
*GR*	Glucocorticoid receptor	(+) agcttcaggatgtcattacggg (−) gagcttcaaggttcattccagc		
*MR*	Mineralocorticoid receptor	(+) gtctgccatgtatgaactgtgc (−) tcctcatctcctcaaatgcagc		
*CNR1*	Cannabinoid receptor 1	(+) aacagcaccactaacatcatgc (-) tgatctgtaaaccactgcttcg		
*GABRA1*	Gamma-aminobutyric acid type A receptor subunit alpha1	(+) ggtttattgcagtgtgctatgc (-) gggtatagctggttgctgtagg		
*MAOA*	Monoamine oxidase A	(+) gccaggaacggaaatttgtagg (−) ttggtttctctcaggtggaagc		
*DAT1*	Dopamine transporter gene 1	(+) ttctgcgtcaccaacggtggc (−) ggacactgccctgaatctgtgc		

### 2.5 Statistics

IBM® SPSS® Statistics Version 27 for Mac was used for all statistical analyses. Three-way ANOVAs were run with Sex (male, female), Stress (control, MS), and Injury (sham, mTBI, surgery) as factors. Post-hoc pairwise Bonferroni corrections were run where necessary for interaction effects, with a *p* value < 0.05 considered statistically significant. All data is available at the opensource framework (OSF) repository: https://osf.io/jbxsz/?view_only=44f00473e970436390f42985ead7e1e2.

## 3 Results

### 3.1 Sex influenced anxiety-like behaviour and surgery increased sensitivity to mechanical nociception

The duration of time spent in the open arms of the EPM was significantly different between males and females (F_(1,87)_ = 11.782, *p* < 0.001), although a significant interaction between sex, stress, and injury was also observed (F_(2,87)_ = 3.317, *p* = 0.041). Post-hoc analyses showed that sex differences were largely driven by two groups. Relative to the males, females spent longer in the open arms in the control sham groups (*p* = 0.002), and in the MS mTBI groups (*p* = 0.009) (Fig. 1B).

In the von Frey task there was a significant main effect of injury (F_(2,92)_ = 33.999, *p* < 0.001) on the filament weight required to elicit a reaction. Post-hoc comparisons revealed that filament weights were significantly reduced following surgery (*p* < 0.001), but not mTBI, when compared to sham groups. There were no significant effects of stress (F_(1,92)_ = 0.848, *p* = 0.360), or sex (F_(1,92)_ = 1.067, *p* = 0.304) on filament weight (Fig. 1C).

### 3.2 mTBI and surgery induced changes to serum CGRP and substance P levels

**Fig. 2:**
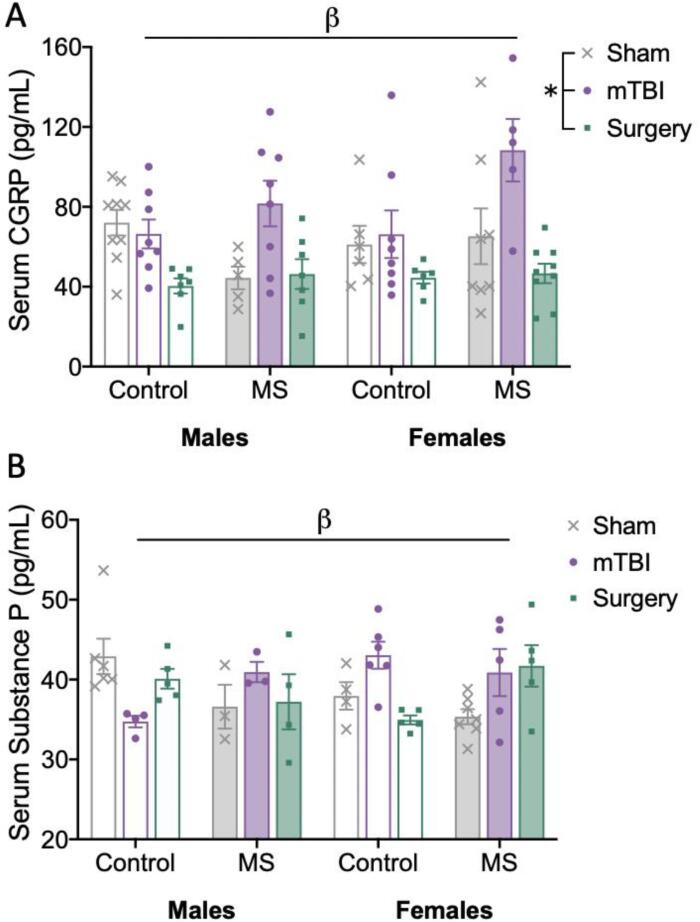
Bar graphs displaying results for serum ELISAs. Means and individual data points ± standard error are shown. (*) indicates a main effect of injury, and (β) indicates a significant interaction; *p’s* < 0.05. A) Serum levels of CGRP, whereby the interaction observed was stress*injury, B) Serum levels of Substance P, whereby the interactions observed were stress*injury and sex*stress*injury.

There was a significant effect of injury (F_(2,75)_ = 15.200, *p* < 0.001) on ELISA quantification of serum CGRP levels. A significant interaction between stress and injury (F_(2,75)_ = 4.275, *p* = 0.017) indicated that the effect of injury differed depending on prior exposure to MS. Post-hoc analyses revealed that in the control groups, surgery reduced serum CGRP levels relative to sham (*p* = 0.02) and mTBI groups (*p* = 0.012); while in the MS groups, mTBI increased serum CGRP levels relative to the sham and surgery groups (*p’s* < 0.001). There were no significant effects of stress (F_(1,75)_ = 2.027, *p* = 0.159) or sex (F_(1,75)_ = 1.274, *p* = 0.263) on serum CGRP levels (Fig. 2A).

For serum levels of Substance P, no main effects of injury (F_(__2,45__)_ = 0.750, *p* = 0.478), stress (F_(1,45)_ = 0.020, *p* = 0.889), or sex (F_(1,45)_ = 0.038, *p* = 0.847) were observed. However, there was a significant interaction between sex, stress, and injury (F_(2, 45)_ = 4.884, *p* = 0.012). Post-hoc analyses revealed that for females exposed to MS, mTBI (*p* = 0.036) and surgery (*p* = 0.016) increased Substance P levels when compared to shams, an effect not observed in control groups or males exposed to MS (Fig. 2B).

### 3.3 Sex, but neither injury nor MS, influenced cerebellar expression of IL1β and GFAP

**Fig. 3:**
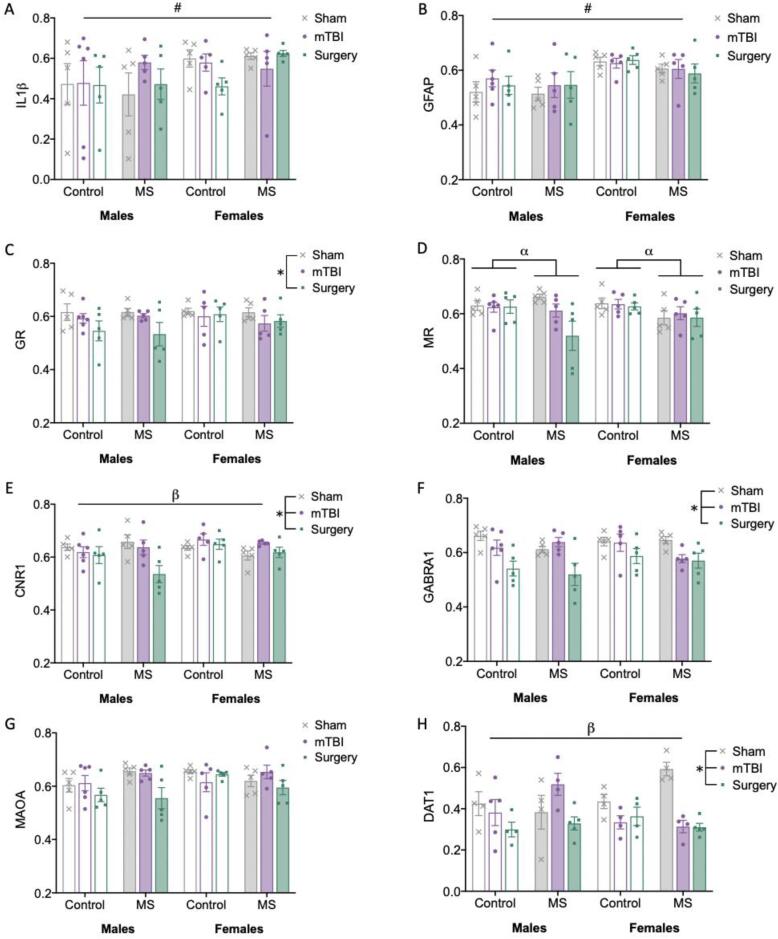
Bar graphs displaying results for cerebellar RT-qPCR gene expression. Means and individual data points ± standard error are shown. (#) indicates a main effect of sex, (*) indicates a main effect of injury, (α) indicates a main effect of stress, and (β) indicates a significant interaction; *p’s* < 0.05. A) Cerebellar expression of *IL1*β; B) Cerebellar expression of *GFAP*; C) Cerebellar expression of *GR*; D) Cerebellar expression of *MR*; E) Cerebellar expression of *CNR1*, whereby the interaction observed was sex*injury; F) Cerebellar expression of *GABRA1*; G) Cerebellar expression of *MAOA*; and H) Cerebellar expression of *DAT1*, whereby the interactions observed were sex*injury and sex*stress*injury.

RT-qPCR was used to quantify gene expression in the cerebellum following injury and MS. Cerebellar expression of *IL1β* was higher in females than in males (F_(1,49)_ = 4.308, *p* = 0.043), but was not significantly influenced by injury (F_(2,49)_ = 0.296, *p* = 0.745) nor stress (F_(1,49)_ = 0.603, *p* = 0.441, Fig. 3A). Similarly, cerebellar expression of *GFAP* was elevated in females relative to males (F_(1,49)_ = 17.289, *p* < 0.001) and there were no significant effects of injury (F_(2,49)_ = 0.341, *p* = 0.713) nor stress (F_(1,49)_ = 1.328, *p* = 0.255, Fig. 3B).

### 3.4 Injury and MS influenced cerebellar expression of genes relevant to stress, inhibition, cannabinoid, and dopaminergic function

There was a significant effect of injury on cerebellar expression of *GR* (F_(2,49)_ = 3.315, *p* = 0.045), but no effects of stress (F_(1,49)_ = 0.391, *p* = 0.535) or sex (F_(1,49)_ = 1.010, *p* = 0.320, Fig. 3C). Post-hoc analysis indicated that surgery (*p* = 0.039), but not mTBI, significantly lowered cerebellar *GR* expression in comparison to shams. *MR* expression in the cerebellum was significantly reduced following MS when compared to control groups (F_(1,49)_ = 5.883, *p* = 0.019), but did not differ based on injury (F_(2,49)_ = 2.545, *p* = 0.089) or sex (F_(1,49)_ = 0.000, *p* = 0.995, Fig. 3D).

A main effect of injury on *CNR1* expression was evident (F_(2,49)_ = 3.945, *p* = 0.026), an outcome that was sex-dependent as confirmed by a significant interaction between sex and injury (F_(2,49)_ = 4.308, *p* = 0.019, Fig. 3E). Post-hoc analysis revealed that injury effects were only present in the males, with surgery resulting in diminished *CNR1* expression in comparison to shams (*p* = 0.001).

Cerebellar expression of *GABRA1* was significantly influenced by injury (F_(2,49)_ = 12.830, *p* < 0.001). Post-hoc analysis showed that surgery significantly lowered *GABRA1* expression in comparison to sham groups (*p* < 0.001), while mTBI groups were not significantly different from sham groups. No effects of stress (F_(1,49)_ = 1.987, *p* = 0.165) or sex (F_(1,49)_ = 0.624, *p* = 0.433) were evident (Fig. 3F).

*MAOA* expression was altered by injury (F_(2,49)_ = 3.896, *p* = 0.027), but not by stress (F_(1,49)_ = 0.139, *p* = 0.711) nor sex (F_(1,49)_ = 2.648, *p* = 0.110, Fig. 3G). Post-hoc comparisons revealed no significant differences between injury groups. *DAT1* expression in the cerebellum was significantly different between injury groups (F_(2,39)_ = 8.324, *p* < 0.001), however a significant interaction between sex, stress, and injury was also evident (F_(2,39)_ = 4.015, *p* = 0.026, Fig. 3H). Post-hoc analyses showed that effects were only significant in female MS groups, where females with prior exposure to MS showed reduced *DAT1* following mTBI (*p* < 0.001) and surgery (*p* < 0.001) in comparison to sham groups.

## 4 Discussion

Chronic pain often produces sexually-dimorphic symptomologies, recovery trajectories, and therapeutic responses following an acute pain trigger, with an increased risk for individuals that have experienced early life stress (King et al., 2011, Eisenberg et al., 2014, Rabbitts et al., 2017, Green et al., 2011). Pain is a highly complex experience, with sensory, affective, and cognitive features processed across varying brain regions (Brooks and Tracey, 2005, Apkarian et al., 2005, Hashmi et al., 2013). Recent evidence suggests that the cerebellum is involved in many of these processes within the context of pain (Moulton et al., 2010). Therefore, our study aimed to examine sex-dependent cerebellar gene expression and chronic pain outcomes that developed following exposure to early life stress and/or an injury. We found increases in nociceptive sensitivity in the surgery group, with surgery animals demonstrating lower mechanical thresholds than mTBI and sham groups. Interestingly, the behaviour tests were completed at a chronic timepoint (>30 days post-injury), suggesting that the deficits persist chronically, beyond what would have been considered typical healing time. Considering the high prevalence of co-morbid psychiatric symptomology that occurs in the context of chronic pain conditions (Means-Christensen et al., 2008, de Heer et al., 2014), we sought to assess anxiety-like behaviour following MS and injury. We found no main effects of MS or injury on anxiety-like behaviour. However, in line with our recent studies, we found that males displayed heightened anxiety-like behaviour relative to females (Salberg et al., 2023, Salberg et al., 2023). Specifically, interaction effects showed that this observation was mostly applicable in the control/sham and MS/mTBI conditions. Although females typically show higher susceptibility to anxiety disorders, we have previously described that increased anxiety-like behaviour in males may be associated with increased systemic inflammatory molecules not observed in females (Salberg et al., 2023). Furthermore, studies demonstrate that early life stress in rodents results in prolonged activation of the HPA axis, which can lead to abnormalities in the development and function of the neonatal central nervous system, thereby resulting in lasting changes to the stress response and emotional function (Nishi et al., 2014, Teicher et al., 2006). Therefore, it is not surprising that anxiety-like behaviour was altered in our rats exposed to neonatal MS, when this was combined with the mTBI. Although we found that females displayed less anxiety-like behaviour than males following this combined MS/mTBI, this is in agreement with a recent study that showed distinct changes to hippocampal function and behaviour between female and male mice following MS (Talani et al., 2023). In particular, our improved anxiety-like outcomes in females relative to males following MS/mTBI corroborates a study by Telani et al, demonstrating that the administration of exogenous estrogens to male mice had protective effects against MS-induced behavioural impairments (Talani et al., 2023).

Of importance, our results indicate that cumulative stressors were required to increase systemic pain markers. Consistent with literature demonstrating positive correlations between the number of adverse childhood experiences and poorer health outcomes (Felitti et al., 1998), the MS/mTBI groups had significantly elevated CGRP levels, while females in the MS injured groups had elevated levels of Substance P. CGRP and Substance P are released following a pain response, which results in vasodilation and inflammation, transmission of nociceptive information, and the perpetuation of pain responses (Durham, 2006, Graefe and Biochemistry, 2022). Given that CGRP is often elevated during migraine attacks in both clinical and preclinical populations, our increase in the mTBI group is in line with prior literature (Durham, 2006, Wattiez et al., 2019). Additionally, Substance P levels were most significantly changed in females exposed to cumulative stressors. Although human and animal studies demonstrate that stressors (Ebner et al., 2004), TBI (Donkin et al., 2007), and chronic pain (Abbadie et al., 1996, Lisowska et al., 2016) are all associated with elevations in Substance P levels, sex differences exist in Substance P levels. For example, gonadal hormones play a role in its regulation with baseline Substance P being positively correlated with estrogen in rats (Duval et al., 1996), and levels of Substance P restored in ovariectomized females with the supplementation of oestradiol (Nazarian et al., 2014). In conjunction with findings from intraplantar formalin injection-induced pain in females (Nazarian et al., 2014), our results suggest that Substance P may be a better indicator of persistent pain in females than in males.

We identified similar findings in inflammatory (*IL1* β) and astrocyte (*GFAP)* markers, whereby levels were increased in females compared to males. This is particularly interesting as levels of inflammatory and microglial markers in other brain regions and systemically are elevated in males but not females (Salberg et al., 2023). These sex differences may manifest because the innate immune response is believed to drive pain in males, whereas the adaptive immune response drives this chronification in females (Sorge et al., 2015).

Corticosteroid receptors including GR and MR are readily implicated in the adrenal stress response and moreover, altered central expression of *GR* and *MR* have been associated with changes to nociceptive sensitivity (Myers and Greenwood-Van, 2009, Johnson and Greenwood-Van, 2015, Ratka et al., 2008). In this study, we identified an association between cerebellar *GR* function and pain, noting that expression of *GR*, but not *MR*, was dampened in rats that underwent surgery and displayed increased sensitivity to mechanical nociception. In the amygdala, heightened mechanical nociception has similarly been associated with the knockdown of GRs (Johnson and Greenwood-Van, 2015), consistent with what we observed in the cerebellum. Interestingly, cutaneous nociception does not appear to be influenced by MR activity in the amygdala (Myers and Greenwood-Van, 2009, Johnson and Greenwood-Van, 2015), a finding which may be relevant as to why we did not see any differences in expression of *MR* in the cerebellum between injury groups, even in the presence of nociceptive differences. Despite this, we noted significant reductions in *MR* following MS, which is consistent with a study that detailed reductions to *MR* in the hippocampus following a MS paradigm in marmosets (Arabadzisz et al., 2010). In contrast, male rodents show evidence of increased hippocampal *MR* expression following early life stress, while female mice showed no changes (Ladd et al., 2004, Bonapersona et al., 2019). Here, we saw no influence of sex on cerebellar *MR*, thereby highlighting discrepancies in *MR* expression between the cerebellum and other brain regions after early life stress. With this said, we have outlined an association between molecular function in the cerebellum and pain, noting patterns of *GR* and *MR* expression in the cerebellum relating to nociception which align with associations demonstrated in other pain-processing regions.

We have also extended the link between the cerebellum and pain to involve cannabinoid function, demonstrating that cerebellar expression of *CNR1*, the gene encoding for the cannabinoid receptor 1 (CB1R), was reduced in males who exhibited increased nociceptive sensitivity following surgery. This was not surprising, given the evidence for an anti-nociceptive and anti-inflammatory function of the CB1R (Clayton et al., 2002, Palazzo et al., 2010). Although we did not see any effects of surgery on *CNR1* expression in females, this aligns with prior findings that show disparate analgesic responses to cannabinoids between male and female rats (Blanton et al., 2021, LaFleur et al., 2018). In a rat model of chronic inflammatory pain, a selective CB1R agonist showed 30-fold higher anti-nociceptive potential in males relative to females (Niu et al., 2012) which, together with our findings, indicate that CB1R function in nociception varies depending on sex. Interestingly, we observed no influence of MS on cerebellar *CNR1* expression, despite previous studies reporting changes to CB1R expression in other brain regions including the hippocampus, amygdala, and prefrontal cortex following stress induced via MS or tail shock (Xing et al., 2014, Viveros et al., 2009). This contrast may be a result of several differing factors such as the time point of CB1R expression analysis, brain regions examined, and models of stress. Nevertheless, we have reported a suppression of *CNR1* expression in the cerebellum of males following surgery. Interestingly, this may be relevant to their reductions in nociceptive sensitivity, as suggested previously (Poznanski et al., 2022). Considering the importance of cerebellar CB1Rs in regulating synaptic plasticity of Purkinje cells, surgery-associated increases in nociception may be associated with CB1R-mediated changes to Purkinje cell function. Given that Purkinje cell activity directly modulates outputs from the deep cerebellar nuclei, as well as the vast efferent projections from the cerebellum onto higher cortical and subcortical regions with nociceptive influence (Moulton et al., 2010, Kang et al., 2021), alterations in Purkinje-cell function may modulate other pain-processing regions.

Furthermore, Purkinje cells possess a large number of post-synaptic and extrasynaptic GABA receptor sites, and are powerfully influenced by GABA released from various interneurons in the cerebellar cortex (Fritschy and Panzanelli, 2006). *GABRA1* encodes for the α1 subunit of the GABA_A_ receptor, and in examining its expression in the cerebellum, it was diminished following surgery, but not altered by stress or sex. Unlike granule cells and interneurons which express GABA_A_ receptors of various subunits, Purkinje cells exclusively express the α1 subtype and are thus more sensitive to changes in α1 subunit function (Fritschy and Panzanelli, 2006). Consequently, a suppression of GABAergic modulation to Purkinje cells, and ultimately cerebellar output, may be a mechanism by which reductions in cerebellar *GABRA1* relate to increased mechanical nociception following surgery.

The cerebellum also receives widespread modulatory innervation from monoaminergic fibres (Panagopoulos et al., 1991, Hökfelt and Fuxe, 1969), and although we did not observe any significant differences between experimental manipulations in cerebellar expression of monoamine oxidase A (*MAOA)*, the dopamine transporter gene (*DAT1)* was reduced in female rats after exposure to combined MS and injury. Accordingly, previous studies have documented changes to dopamine transporter function associated with prolonged stress or TBI in animal models (Isovich et al., 2000, Novick et al., 2011, Yan et al., 2002, Wilson et al., 2005), though these studies only included males. Moreover, genetic differences in *DAT1* between humans with divergent tolerances to cold pain has been recognised (Treister et al., 2009). Interestingly, we did not see cerebellar *DAT1* changes in females that were only exposed to a single stressor, implying that the combined effects of MS and injury were necessary to prompt cerebellar *DAT1* changes. Furthermore, the stress and injury conditions did not significantly change cerebellar *DAT1* in males, illustrating a sexually dimorphic response. Taken together, we have noted reductions in cerebellar *DAT1* for a subgroup of rats displaying post-surgical increases in mechanical nociception, specifically in females with prior exposure to MS. Although cerebellar dopaminergic function may be related to nociception, this association is less apparent here, given there was a lack of *DAT1* change in other subgroups of rats exhibiting heightened post-surgical nociceptive sensitivity.

## 5 Conclusion

We have illustrated that in female and male adolescent rats, mechanical nociceptive sensitivity increased following surgery, while remaining unchanged after early life stress. We did not observe sex differences in mechanical nociception however, we demonstrated that males displayed higher anxiety-like behaviour than females, specifically following early life stress and mTBI. Notably, we demonstrated that rats presenting with heightened mechanical nociception displayed suppressed expression of genes in the cerebellum that regulate stress, inhibitory neurotransmission, cannabinoid function, and dopaminergic function, alongside sex-dependent distinctions for inflammatory and injury markers. In doing so, we highlight a novel link between nociception and molecular function in the cerebellum. We acknowledge that this is an association which has not been explicitly addressed but nevertheless, this reinforces the hypothesis that the cerebellum is involved in the chronification of pain. As such, future studies should continue to explore this relationship, perhaps seeking out additional models of pain, and extending analyses of cerebellar function to include histology, protein expression, or neuroimaging. Unquestionably, further investigations into how the cerebellum plays a part in pain within males and females could facilitate novel therapeutic insights and opportunities.

## CRediT authorship contribution statement

**Sabrina Salberg:** Methodology, Validation, Formal analysis, Investigation, Data curation, Writing – original draft, Writing – review & editing. **Crystal N. Li:** Methodology, Validation, Formal analysis, Investigation, Data curation, Writing – original draft, Writing – review & editing. **Jaimie K. Beveridge:** Writing – review & editing. **Melanie Noel:** Conceptualization, Writing – review & editing, Supervision. **Glenn R. Yamakawa:** Methodology, Data curation, Writing – review & editing. **Richelle Mychasiuk:** Conceptualization, Methodology, Formal analysis, Resources, Writing – review & editing, Visualization, Supervision, Project administration, Funding acquisition.

## Declaration of Competing Interest

The authors declare that they have no known competing financial interests or personal relationships that could have appeared to influence the work reported in this paper.

## Data Availability

The data will all be available at open source framework upon publication of the manuscript
